# The Use of Nanoscale Montmorillonite (MMT) as Reinforcement for Polylactide Acid (PLA) Prepared by Fused Deposition Modeling (FDM)—Comparative Study with Biocarbon and Talc Fillers

**DOI:** 10.3390/ma15155205

**Published:** 2022-07-27

**Authors:** Jacek Andrzejewski, Mateusz Markowski, Mateusz Barczewski

**Affiliations:** 1 Polymer Processing Division, Institute of Materials Technology, Faculty of Mechanical Engineering, Poznan University of Technology, Piotrowo 3 Str., 61-138 Poznan, Poland; mateusz.barczewski@put.poznan.pl; 2 MATRIX Students Club, Polymer Processing Division, Institute of Materials Technology, Poznan University of Technology, Piotrowo 3 Str., 61-138 Poznan, Poland; mateusz.m.markowski@student.put.poznan.pl; 3 Faculty of Materials Engineering and Technical Physics, Poznan University of Technology, Piotrowo 3 Str., 61-138 Poznan, Poland

**Keywords:** fused deposition modeling, materials performance, polymer fillers, polylactide acid

## Abstract

The subject of the presented research focuses on a comparative assessment of three types of polymer fillers used to modify highly crystalline poly(lactic acid) PLA intended for the FDM technique. The aim of the presented work was to determine the performance of the developed materials. The key aspect of the work was the use of polymer fillers of three different types. Nano-sized montmorillonite (MMT), biobased biocarbon (BC) and mineral talc. The several types of composites were prepared using extrusion technique. The maximum content for BC and talc filler was limited to 20 wt%, while for MMT it was 5 wt%. Prepared samples were subjected to detailed material analysis including mechanical tests (tensile, flexural, Charpy), thermal analysis (DSC, DMTA), HDT/Vicat tests and structure analysis. The results of the test confirmed that even relatively small amount of nano-type filler can be more efficient than micrometric particles. The used type of matrix was highly crystalline PLA, which resulted in a significant nucleation effect of the crystalline structure. However, thermomechanical tests revealed no improvement in thermal resistance. Microscopic survey confirmed that for MMT and talc filler the structure anisotropy was leading to more favorable properties, especially when compared to structures based on spherical BC particles.

## 1. Introduction

Three-dimensional printing is the process of creating three-dimensional objects in layers. In contrast to subtractive technologies, such as drilling, milling, sawing, etc., additive technologies enable the production of highly complex parts, which gives almost endless possibilities for rapid prototyping. The advantages of additive production include: great freedom in design, quick production of ready-made models, flexibility and the ability to produce complex designs and high durability [1]. The greatest advantage, however, seems to be the possibility of using almost any type of material—from polymers, through metals, to ceramics. The development of technology has made it possible to produce faster, cheaper and, above all, smaller printing devices. Over time, many new 3D printing techniques have been developed. However, due to the low costs of materials and devices, the most popular 3D printing technique today is the FDM method. At present, numerous modifications of the FDM technique are being developed, enabling the printing of plastics reinforced with continuous fibers [2,3,4,5,6] or highly filled composites intended for further sintering processes [7,8,9,10,11]. Due to the fact that devices operating in new variants of the FDM technique require numerous modifications, these technologies are not widely used mainly due to the high cost of the devices. A more optimal solution is to modify the composition of the filament, most often by addition of fillers or using polymer blends. Unlike for the injection molding technique, the use of fibrous reinforcement during FDM printing is less favorable since the increase in viscosity restricts flow through a small printer nozzle. The use of spherical fillers is less problematic, but the processing of highly filled materials as filament is not recommended [12,13]. 

An interesting example of the use of mineral fillers in FDM printing are biomedical applications including the production of bioresorbable implants intended for bone tissue reconstitution. In many applications the problem is the required high content of CaP-based fillers for bone-forming materials, which is a serious limitation in the FDM technique. Another segment of research devoted to the polymer filler of the FDM technique includes works devoted to the use of nanoadditives. Thus far, dozens of studied were carried out on this type of fillers concerned almost all types of nanometric materials, including nanoclays, nanocellulose and all types of carbon-based additives such as graphene or nanotubes. Previous research almost always indicates an improvement in material properties, but usually the reference point is pure, unmodified polymer resin, which is why in the presented research we focused on identifying the fundamental differences between the addition of nanofillers and micrometric particles. This approach may help in a wider assessment of the rationale behind the use of nanomaterials. 

The presented research focuses on the modification of PLA with fillers, which in the case of the discussed issues of 3D printing is not a significant novelty, because many previous works have discussed this topic [14,15,16]. However, unlike previous works, the currently used polymer constituting the composite matrix is characterized by a high degree of crystallinity; while thus far most of the PLA varieties used in the additive technique are characterized by low crystalline phase content. High-crystalline varieties of PLA are currently used for processing injection molding; a technique by which materials with high mechanical and thermomechanical resistance can be obtained under appropriate process conditions. The use of polymer fillers to initiate the process of crystal phase growth is very popular for improving the properties of semicrystalline polymers such as PA6 or PP [17,18,19,20], but in the case of materials characterized by a slow crystallization process, it requires the creation of favorable thermodynamic conditions during processing [21,22]. So far, the issue of increasing the content of the crystalline phase of PLA has been the main goal during the processing of injection molding. However, large-volume techniques are characterized by the necessity to use short cycle times, which, even with the use of advanced modifying systems, does not give the desired results. In the case of 3D printing, the production time of a single product is not a technological limitation, therefore it is potentially possible to produce materials in conditions that allow to obtain a high level of crystallinity. 

The presented text is a summary of research work aimed at assessing the properties of PLA after the printing process. The material tests were aimed at assessing the effectiveness of the composite structure strengthening as a result of the use of three different types of polymer fillers. A particularly important aspect of the work is the assessment of thermomechanical properties in correlation to the results of calorimetric measurements, where the properties of the printed samples were assessed in terms of the content of the crystalline phase.

## 2. Materials and Methods

### 2.1. Materials

The polymeric materials necessary for the preparation of the test samples were prepared as a result of a three-stage manufacturing process, including mixing the components on a twin-screw extruder, producing a filament on a single-screw extruder and printing samples using the FDM method. The materials used for the purpose of the study are:

− PLA—Poly(lactic acid) granules, variety Ingeo 2500D (NatureWorks, Minetonka, Minneapolis, MN, USA), MFI = 8 g/10 min (210 °C; 2.16 kg),
− MMT—Montmorillonite nano-clay from Sigma-Aldrich, particles were surface-treated with 0.5–5 wt% aminopropyltriethoxysilane, 15–35 wt% octadecylamine,
− Talc—the talcum clay filler (magnesium hydrogen metasilicate), producer: Biomus,
− BC—Biocarbon powder, obtained during the pyrolysis process at 650 °C, particular filler was prepared from the wood chips. Before using the filler was ball milled for 24 h, the procedure and properties of biochar was described in our previous work [23]. Producer: Fluid S.A.

The list of sample designations and formulations for all materials is listed in the Table 1. When selecting the filler content for micrometric materials (BC and Talc), we decided to prepare materials with a content of 10 and 20%, which can be consider as a relatively large amount for the FDM method, since usually the content of fillers does not exceed 10% [24]. However, for the present study the maximum percentage was increased to 20% so that the effectiveness of the used fillers was more significant while still maintaining sufficient processability. For the MMT nano-additive, we decided to take a slightly different approach: instead of using the same concentrations as for the micrometric additives, we reduced the maximum content to 5%, which can be consider as high MMT content compared to other studies, where PLA/MMT composites were prepared [25,26,27].

### 2.2. Sample Preparation

In the first stage, the PLA pellets were melted and blended with individual fillers on a ZAMAK EH 16.2D twin-screw extruder (Zamak Mercator, Skawina, Poland). The maximum temperature of the extrusion process was 190 °C, and the rotational speed of the screws was 100 rpm. The extrusion rate with these machine parameters was about 2 kg/h. The preparation of the material before the extrusion process involved drying of PLA granules and fillers in a cabin dryer for 12 h at a temperature of 80 °C. Before extrusion, all components of the mixture were placed in a polyethylene zip-bag, tightly closed and vigorously mixed. The dry-blend prepared in this way was placed in the hopper of the twin-screw machine and blended in the molten state. After exiting the extruder head, the extrudate material was cooled in a stream of cold air and continuously pelletized by means of a milling granulator.

The filament preparation stage was performed on a METALCHEM 20–32D single-screw machine (IMPiB, Toruń, Poland). The maximum extrusion temperature set the die-head was 190 °C, while the rotational speed of the screw was set at 10 rpm. The processed granules were dried at 70 °C for 12 h. The extrusion die diameter was 3 mm, while the final filament diameter of about 1.75 mm was controlled by the suction speed, which, depending on the melt viscosity, ranged from 5 to 7 m/min. The filament was cooled in a stream of cold air, while the finished material was wound on spools with a winder.

The printing process was carried out on a Prusa MK3S printer (PrusaResearch, Prague, Czech Republic). For all materials, the die temperature was 215 °C, the bed temperature was also constant for all samples at 60 °C. The printing speed for most samples was about 50 mm/min. G-code files were generated in the Slic3r Prusa Edition program.

### 2.3. Characterization

Mechanical tests were carried out with the use of three measurement methods, static tensile and flexural tests and Charpy impact method. In the case of static tensile measurements, they were carried out on the Zwick/Roell Z010 machine; the crosshead speed was 10 mm/min; and the tests were carried out on samples of type 1A (gauge length 50 mm). The flexural tests were also carried using the same universal testing machine. The bending speed was 2 mm/min, and the supports were 64 mm wide. The Charpy method measurement were carried out using Zwick/Roell HIT25 apparatus. Machine was equipped with 5 J hammer. Tests were conducted using notched specimens with a notch depth of 2 mm. All mechanical tests were conducted in accordance with specific standard, ISO 527 for tensile tests, ISO 178 for flexural tests and ISO 180 for Charpy measurements. The obtained results are the average of a minimum of five measurements.

Thermal properties of prepared materials were evaluated using differential scanning calorimetry method (DSC). Tests were performed on samples weighing around 5 g cut from the material samples. The samples were placed in aluminum crucibles and sealed with a pierced lid. They were then placed in the measuring chamber of the Netzsch DSC F1 204 Phoenix apparatus. The test was carried out in the temperature range of 20–220 °C, at the heating/cooling speed of 10 °C/min. During the analysis, the samples were under a protective nitrogen atmosphere, the flow rate of which was 10 mL/min. The crystallinity level was calculated using the following Formula (1):

(1) $$\%\;Crystallinity=X_{c}=100\times \frac{\Delta H_{m}-\Delta H_{c}}{\Delta H_{100}\;\left(1-\varphi \right)}$$

where ∆*H_m_* is the measured melting enthalpy, ∆*H_c_* is the measured enthalpy of cold crystallization, and Δ*H*_100_ is the theoretical melting enthalpy of 100% crystalline structure. For our study, we used the value of 93.7 J/g [28,29]. The amount of the filler was expressed by φ.

Thermomechanical performance was measured using two methods: dynamic mechanical thermal analysis (DMTA) and HDT/Vicat tests. DMTA measurements were carried out using an Anton Paar MCR 301 rheometer, Anton Paar, Graz, Austria. The apparatus was equipped with torsion mode clamps. The temperature range was from 30 °C to 150 °C with an increase of 2 °C per minute. The distortion frequency was programmed at 1 Hz, and the deformation amplitude value was 0.01%. The thermomechanical analysis in the form of sample deflection under load (HDT) and measurement of the softening point using the Vicat method (VST) was carried out using the Testlab RV300C machine, TestLab, Warszawa, Poland. The load of 0.455 MPa was used in the HDT method, while the VST tests the load was 10N. The heating speed was 2 °C/min in both cases.

The structural assessment was carried out using the scanning electron microscopy (SEM) method with the use of a Zeiss Evo 40 microscope, Carl Zeiss, Jena, Germany. Before the tests, the samples were sputtered with a thin layer of gold on the surface of the throat obtained as a result of previous impact tests. The structure of the samples is shown at ×200, ×2000 and ×10,000 magnification at the breakthrough site. The particle size distribution plots were prepared on the basis of the obtained SEM pictures. The necessary results are obtained from the analysis of around 200 particles, for each filler.

The density of raw materials was measured using helium pycnometer (Thermo Scientific Pycnomatic, Thermo Fisher, Waltham, MA, USA, according to ASTM 794-66 standard). The density of the filament samples and printed specimens was measured using the immersion method according to ISO 12154 standard, and a minimum of five specimens was used for each sample type.

## 3. Results

### 3.1. Composite Structure Evaluation—SEM Observations

**Figure 1:**
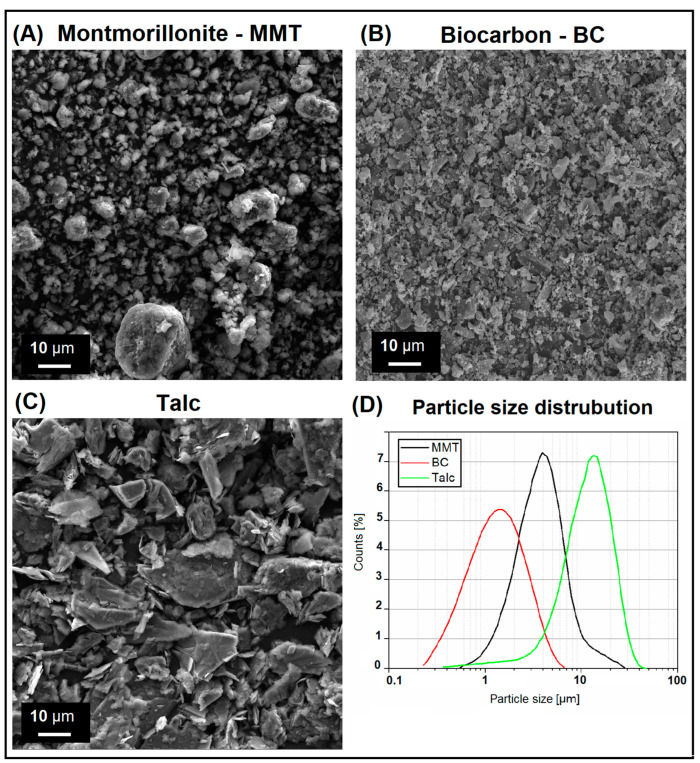
The appearance of (**A**) montmorillonite—MMT, (**B**) biocarbon—BC, and (**C**) talc fillers. (**D**) The size distribution of the presented fillers obtained during SEM pictures analysis.

**Figure 2:**
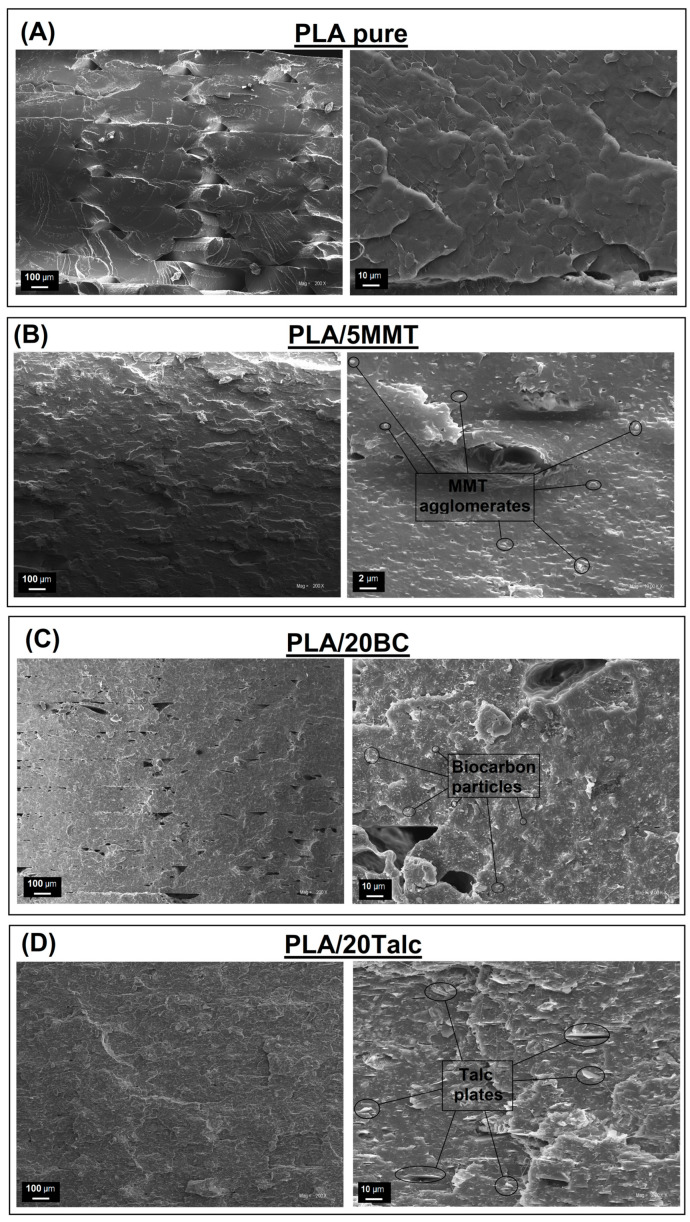
The SEM observations results. Pictures are presenting the fractured surface of (**A**) pure PLA, (**B**) PLA/5MMT (**C**) PLA/20BC and (**D**) PLA/20Talc samples.

The SEM analysis for pure fillers is presented in Figure 1, where all materials are presented at constant magnification for comparison. The composite structures are presented in the Figure 2, where samples with the highest content of individual fillers are compared. The results of the filler particle size measurements are presented in the form of size distribution charts (see Figure 1C). 

The appearance of the filler particles shown in Figure 1 indicates that the particle size for each of the used fillers does not exceed 10 µm. Interestingly, the appearance of the MMT nanofiller would indicate a micrometric nature of this materials. Size distribution plots indicate that the average size of the MMT particles was around 5 µm; however, there are also particles with more than 20 µm in diameter. In this case, the nanometric structure is difficult to identify due to the agglomeration of the particle structure, which are additionally covered with a functional coating made of a mixture of aminopropyltriethoxysilane and octadecylamine. Further structural observations of MMT particles in the PLA matrix indicate the breakdown of agglomerates and a significant reduction in their size. 

In the case of the other two materials, the expectations regarding the particle size are not so high, therefore the average BC particle size of around 1 micron seems to be a promising result. The analysis revealed that the size of talc particles is slightly larger and reaches up to 30 µm, while the average size was close to 10 µm. However, the lamellar (plate) structure of talc is the main advantage of its use as a reinforcement.

The use of fillers in the FDM technique makes it necessary to keep the particle size in the range of several micrometers. Although the nozzle diameter for most 3D printing devices is about 0.5 mm, which is similar to the diameter of the hot runner nozzle gate. The pressures generated during the injection process allow to easily overcome most of the flow resistance, which is impossible during FDM printing due insufficient pressure during the polymer flow. 

The composite structure analysis presented in the Figure 2 consists of pictures presenting the fractured surface of pure PLA (Figure 2A). The appearance of the sample breakthrough revealed more evident roughness than for typical samples prepared from highly amorphous PLA. This behavior proves the increased content of the crystalline phase for the prepared materials, while usually for PLA samples the surface of the sample is smooth [30,31]. For the rest of the samples, the surface roughness is caused by the presence of the fillers. However, as DSC measurements confirmed, the crystallinity of the composite materials is significantly increased compared to pure PLA. In the case of PLA/5MMT samples, it is possible to distinguish the two-phase structure of the material. However, due to the limitations of the SEM technique, the visible layered structure of the sample surface reflects the matrix fracture planes in the vicinity of the filler particles. The spherical particles with a diameter of about 0.5 µm are MMT agglomerates. The appearance of the structure indicates good dispersion of nanoparticles which is not common for other types of nanofillers [32]. Interestingly, the appearance of all composite samples at low magnification indicates an almost solid structure of the sample. Usually for materials prepared by the FDM method the layered nature of the material is very distinct, which can be easily confirmed by the appearance of the pure PLA sample. The observed change may be related to an additional melt blending process on a twin-screw machine. Thermal decomposition leads to PLA decreasing the viscosity. Due to the relatively high viscosity of pure PLA, the printing process was carried out at a temperature of 215 °C, which is relatively high compared to standard PLA based filaments. In this case, for composite samples with a higher rate of matrix degradation, the structure tends to be more compacted since polymer flowing from the nozzle fills the programmed material path more accurately. 

The appearance of the PLA/20BC sample structure indicates a high tendency to brittle cracking which is suggested by the almost flat surface of the specimen fracture. The appearance of the sample at higher magnification revealed a large number of voids, especially between the individual layers of the sample. The structure of the material is more heterogeneous compared to the MMT-based composite. Despite their small size, the BC particles are clearly visible against the matrix background, which proves a low level of compatibility. Contrary to MMT, the surface of BC particles was not modified, which in this case indicates the necessity to use surface modification. Previous studies confirmed the presence of structure self-compatibility for composite systems containing the BC-based fillers [23,33,34,35]. However, the polarity of the PLA chains is too low to form strong bonds with functional groups on the surface of BC. Similar behavior was observed for other thermoplastic polyesters or polyolefins [36,37,38,39,40,41]. The structure of the PLA/20talc composite is very similar to the materials with additional BC. The separation of the talc particles surface from the polymer matrix is clearly visible. A significant change, however, is the clearly lamellar nature of the talc particles and their visible orientation is in line with the direction of polymer flow during the printing process. Orientation during flow is an important feature of talcum-based fillers. The orientation of the composite structure causes a significant increase in mechanical properties which is often used during injection molding, where talc is one of the most commonly used additives [42,43]. Measurements of mechanical properties for the tested samples clearly show the favorable properties of this type of morphology. 

In the FDM technique, the structure of the finished part is built of many layers of overlapping filament paths; therefore, the space between the individual layers of the material and between the filament paths is usually only partially filled with molten material. SEM observations partly confirmed the presence of macro/micro voids within the structure volume, however more valuable information about the void content can be obtained from the density measurements. The results collected in the Table 2 are presenting the theoretical density calculations and the results of density measurements for filament samples and FDM printed parts. Density of powder fillers was measured using helium pycnometer. The density for MMT was 2.46 g/cm^3^, for BC particles 1.65 g/cm^3^ and for Talc filler 2.84 g/cm^3^, and these values were used for calculations.

It is clear that the density of the filament samples was very close to the calculated theoretical value. The porosity for most of the samples was close to 1%, which means that the homogeneity of the prepared materials was at a high level. Larger deviations from the theoretical density values can be noticed for the materials after the FDM printing procedure. The phenomenon of reduced structure density occurs for almost all 3D printing techniques, while for the FDM method, it is often a planned feature. For most types of printing filaments it is recommended that the material flow should be slightly reduced to prevent over-flowing [44,45]. However, in the case of prepared specimens, such an operation did not take place, because we wanted to obtain solid structure. Nevertheless, structure appearance revealed the presence of micro pores, which is visible in the SEM pictures. The difference between the theoretical and measured densities indicated the porosity (void content). 

### 3.2. Mechanical Performance—Static Tensile/Flexural Tests and Impact Resistance Measurements

**Figure 3:**
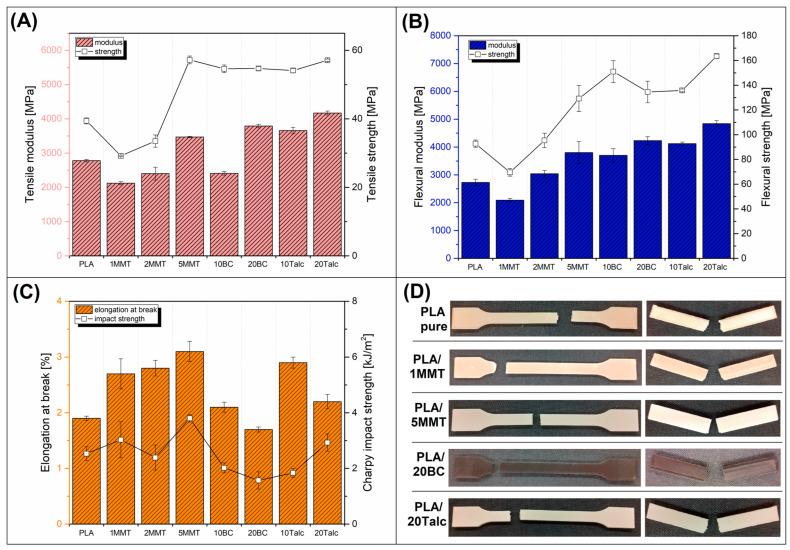
The results of the mechanical properties evaluation. (**A**) tensile modulus and strength; (**B**) flexural modulus and strength; (**C**) elongation at break and impact strength; (**D**) the appearance of samples after testing.

The results presented in this section show the results of all mechanical tests and the appearance of the specimens after testing (see Figure 3). The full table containing the results of mechanical test can be found in the Supplementary Information Section (Table S1).

The performed static tests showed a significant increase in stiffness expressed by tensile modulus and tensile strength in highly filled samples, which is an expected and desired phenomenon. The exception from the general trend is the addition of 10% BC, which negatively affected the stiffness of PLA. However, the deterioration was observed only for tensile modulus, while the flexural modulus and tensile/flexural strength was still higher than the results for pure PLA. The worst strength/modulus results were obtained for materials with a low addition of MMT (1 and 2%). It is worth adding that due to the use of PLA with high crystallinity, the apparent improvement in strength and modulus indirectly show the beneficial changes in the morphology of the crystalline phase, rather than large efficiency of the used fillers [46]. 

The increase in the stiffness of the modified samples had a negative impact on the impact resistance of the already brittle PLA. Two compositions are an exception here. The addition of 20% talc, which significantly increased the stiffness and strength, but also did not lower the toughness of the impact. The second distinctive composition is the PLA blend with the addition of 5% MMT particles. Its presence had a positive impact on every examined mechanical aspect of the material. For other types of fillers, only stiffness and strength were significantly improved, while the elongation and impact resistance were slightly the same or reduced. 

As can be seen in the general view photos (Figure 3), the samples after the tensile fracture are characterized by brittleness, which for PLA-based materials is quite a typical feature. It is worth pointing out that the addition of fillers does not lead to a significant reduction in the elongation value, which is sometimes the case, especially with the addition of spherical particles [47,48]. The appearance of the samples after the impact test confirmed the brittle type of fracture; however, some minor differences in the deformation mechanism can be seen. For samples containing the addition of 20% talc, a slightly greater irregularity of the fracture surface is visible, it is manifested by cracking of individual layers of the filament in several planes, similar to the case of unmodified PLA samples. Such behavior suggests a weakening of the adhesion of individual layers of the product, which in this case may have many causes, related to the increased viscosity of composite samples which limits the layer-to-layer contact surface. The same contact area may be limited due to the presence of particles on the surface of the molten material. In both cases, these phenomena limit the diffusion between the layers of the printed model and lead to its delamination. In the case of the prepared sample models, where the density of the filling used is the highest (infill 100%), such behavior is not of great importance. However, in the case of more complex models made with a partial infill and thin shell layer, such behavior may be the cause of greater susceptibility to cracking. 

Taking into account the observed changes in mechanical properties, it should be indicated that for the obtained composite samples, the most important factor is the relatively high strength value, which proves a high coefficient of structure strengthening. Taking into account the broader comparison, the obtained strength is very close to that obtained for PLA processed by injection molding technique [49,50], which is of high utility value to prototypes made of the materials being developed. As can be seen only for the PLA/20Talc sample, the porosity slightly exceeds 5%, while for the rest of the materials it was below 4%. Such results confirm that the quality of the produced samples is good. Additionally, the low void content indirectly confirmed favorable processability, while the viscosity of the melted material is still at a sufficiently low level and does not limit the polymer flow.

### 3.3. Thermomechanical Properties—DMTA Analysis and HDT/Vicat Tests

The viscoelastic properties of the prepared samples were determined using DMTA analysis, where the results in the form of storage modulus and tan δ plots are collected in the Figure. Thermal resistance tests are complementing the results of the thermal analysis for all samples. Results of HDT/VST measurements are presented in the Table 3—the same table collect the calculated reinforcing C factor.

**Figure 4:**
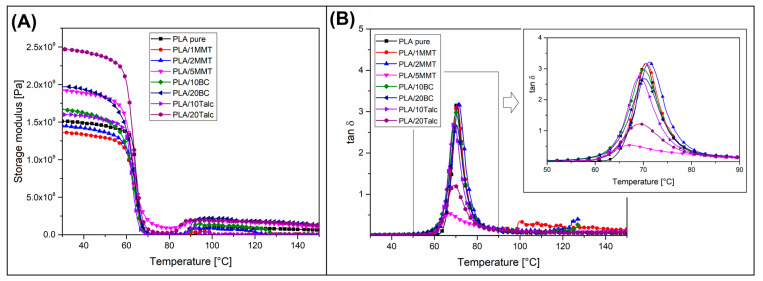
The results of the DMTA analysis. (**A**) storage modulus plots; (**B**) tan δ plots.

The storage modulus comparison, presented in the Figure 4A, confirmed that for some samples, the addition of fillers was not improving the material’s stiffness. For PLA/1MMT and PLA/2MMT composites the until vales of the modulus, recorded below the glass transition region, are even lower than for the reference PLA. More favorable changes are reported for samples with addition of 10% of the BC and Talc fillers; however, still the magnitude of the storage modulus increase is small, since more significant changes are observed for samples with the highest content of the reinforcing particles (20%). The greatest stiffness was recorded for the PLA/20Talc sample, while the storage modulus values for PLA/20BC composite were visibly lower. The most interesting behavior was reported for the PLA/5MMT sample: the storage modulus values are very close to the results obtained with addition of 20% BC. This behavior clearly confirmed that the reinforcing efficiency for nanosized MMT is high. After reaching the T_g_ region, the storage modulus values drop sharply; however, for PLA/5MMT material, the appearance of the plot suggests that the stiffness in rubbery state was significantly higher than the other samples. That behavior suggests that for MMT reinforced samples the matrix-filler interactions are stronger than for standard powder fillers. 

The presented DMTA analysis comprises also the tan δ plots comparison (see Figure 4B). It is clear that for samples containing a small number of fillers, like PLA/1MMT and PLA/2MMT, the T_g_ peak appearance is very similar to the reference PLA. The first noticeable changes are revealed for samples containing 10% of the filler (BC and Talc). Additionally, it can be seen that for the sample PLA/20BC the changes are also relatively small. The magnitude of the changes confirms the presence of fillers in the polymer structure rather than the increased amount of polymer chains entangled at the filler-polymer interface. A significant level of changes is noticeable after the addition of 20% talc, where the area under the tan δ peak is strongly reduced. Even more evident changes are observed for PLA/5MMT samples, where, in addition to the largest change in the areas under the plot, a significant shift in the peak maximum is also observed. The glass transition peak for the reference PLA was recorded at 70.5 °C. For most of the sample, the deviation from this value does not exceed 1.5 ° C. For the PLA/5MMT sample, the T_g_ was observed at 67 °C. The presence of this type of change clearly indicates a high level of structure strengthening for composites modified with MMT particles. However, it is necessary to use a sufficiently high content of the filler. It is worth pointing that usually for FDM printed PLA-based materials the crystallinity level is relatively low, which is mainly related to the tendency to use slowly crystallizing varieties of this polymer [30,51]. In our study we used the highly crystalline type of PLA resin, which also means that the addition of fillers may increase the content of the crystalline phase. From that point of view, it is possible that the observed difference in reinforcing efficiency may be related to the increasing level of crystallinity rather than the intensity of filler-matrix interactions. 

The thermomechanical analysis is supplemented by HDT/Vicat tests, which results are presented in the Table 3. Compared to more complex DMTA test results, the results of basic thermal resistance tests are expressed by a single temperature indicating the deflection of the beam specimen (for HDT) and needle indentation (for Vicat). Although this type of information does not allow for the assessment of structural changes within the performed modifications, they nevertheless allow for a very reliable verification of material application restrictions. For the prepared composites the obtained results suggest lack of large difference between the examined samples; however, there is still a relatively large improvement compared to other types of PLA-based FDM printed samples. Our previous study [51], indicated that for low crystalline PLA resin the HDT results are visibly lower, around 55 °C. It is clear that for highly crystalline materials used during this study the thermal resistance should be more favorable. The initial HDT temperature for pure PLA samples reached 62 °C, which can be consider as quite a good result for the printed sample. Results for composite samples are less favorable than expected, since for PLA/1MMT, PLA/10BC and PLA/10Talc, samples that obtained HDT results did not exceed 60 °C. A partial explanation for that kind of behavior could be the relatively low concentration of the used fillers; however, for other materials containing 20% BC/Talc and 5% MMT, the HDT results were also close to the reference values. The results of the HDT measurement are in line with the Vicat test. Due to the different methodology of the measurement, the Vicat temperature results are slightly higher than HDT. However, it is still clear that highest thermal resistance was recorded for PLA/5MMT, PLA/20BC and PLA/20Talc, while still comparing to reference PLA (VST = 68.1 ± 0.9 °C) the best result was only 5 °C higher. Results confirmed that for FDM printed samples the use of particle type of filler is not leading to large improvement of the thermal resistance. Like for other shaping techniques the use of fibrous reinforcement is more effective for PLA-based composites, which was already confirmed in previous studies [52,53,54].

Apart from the standard mechanical and thermomechanical tests, the general performance of the developed composites is possible to be evaluated by multifactorial analysis. For this purpose, we used the C factor calculations, also called composite performance coefficient. This kind of approach has already been presented in many other publications [55,56,57]. The calculations are based on storage modulus analysis, and expressed by following Formula (2):

(2) $$C\;factor=\frac{{\left(E_{g}^{\prime }/E_{r}^{\prime }\right)}_{composite}}{{\left(E_{g}^{\prime }/E_{r}^{\prime }\right)}_{matrix}}$$

where the $E_{r}^{\prime }$ and $E_{g}^{\prime }$ values are collected from the storage modulus curves in glassy and rubbery region. In this particular example, the C factor was calculated from the storage modulus values at −25°C and at 80 °C. The results of the performed calculations revealed large differences in reinforcing efficiency for the used fillers. For conventional talc filler the addition of 10% was leading to a relatively small effect, even compared to BC filler. A possible reason for this phenomenon is a slightly lower volume content of talc filler compared to BC, which resulted from the difference in density of these materials; 1.3 g/cm^3^ for BC and 2.7 g/cm^3^ for talc respectively [58]. The addition of 20% talc significantly improves the effectiveness of the reinforcement, which clearly suggests a decrease in the C-factor index. The same coefficient for PLA/20BC sample was only slightly reduced, which confirmed less effective reinforcement. As the results show, MMT particles are the most effective type of filler. Although for the sample containing 1 and 2% of nanosilica, the structure strengthening does not occur or is very small, the increase in MMT content to 5% already significantly improved most of the mechanical characteristics. From a scientific point of view, higher amounts of MMT could be used possibly resulting in even more marked changes in the mechanical characteristics. However, taking into account the nanoadditives’ tendency to agglomerate, increasing the content would lead to a decrease in the effectiveness of the structure strengthening, which, combined with the high cost of nanometric particles, is not an optimal solution.

**Table 3:** The results of heat deflection temperature (HDT), Vicat softening point (VST) measurements, and C-factor calculations.

Sample	HDT (0.455 MPa) [°C]	VST * (10 N) [°C]	C Factor [–]
PLA	62.0 ± 0.3	68.1 ± 0.9	1.00
PLA/1MMT	57.3 ± 0.8	67.3 ± 2.1	1.10
PLA/2MMT	61.7 ± 0.1	69.4 ± 0.1	0.81
PLA/5MMT	62.2 ± 0.9	73.6 ± 4.0	0.03
PLA/10BC	57.8 ± 0.2	66.4 ± 0.6	0.77
PLA/20BC	63.4 ± 0.4	72.1 ± 0.3	0.64
PLA/10Talc	59.5 ± 1.1	67.0 ± 3.4	0.95
PLA/20Talc	60.7 ± 0.3	69.8 ± 0.1	0.13

* VST—Vicat softening temperature.

### 3.4. DSC Analysis

**Figure 5:**
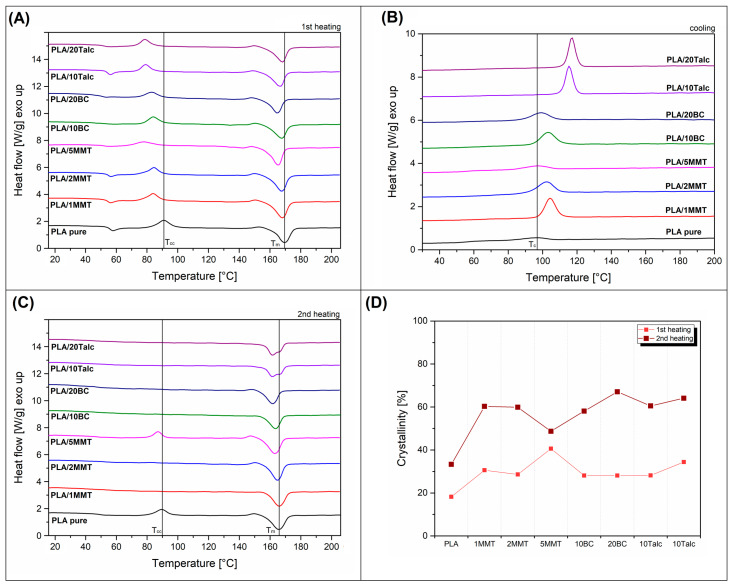
The results of the DSC analysis. (**A**) 1st heating; (**B**) cooling; (**C**) 2nd heating; (**D**) PLA crystallinity.

The summaries presented in the section below include the results of the performed DSC measurements. DSC thermograms are collected separately for 1st heating, cooling and 2nd heating stage of the measurements (see Figure 5A–C). The last of the graphs reveal the results of calculations of the degree of crystallinity for individual samples. The basic data obtained during the analysis of the DSC plot are collected in the Table S2 (Supporting Information Section).

The 1st heating thermogram comparison revealed the typical behavior of slowly crystallizing polyesters, where the endothermic melting peak is preceded by a smaller exothermic peak reflecting the phenomenon of cold crystallization of PLA. For pure PLA, the cold crystallization peak appears at around 92 °C while for all composite materials the maximum was shifted to lower temperatures. For talc-based composites and PLA/5MMT samples, the change of T_cc_ was highest as the peak position was below 80 °C. This behavior indicates changes in the crystallization kinetics for composites and suggests an increase in the intensity of crystal phase nucleation due to the presence of filler particles. Despite some shifts in the peak position, the appearance and area under the T_cc_ peak is almost similar for most of the samples. The exception here is the PLA/5MM sample, where the reduced area under the peak suggests more significant structural changes. The melting of the crystalline phase for all of the samples takes place above 150°C. For pure PLA the melting peak was recorded at 169 °C, while for the other samples it is shifted by a maximum of 4 degrees. 

The enthalpy measurements made it possible to calculate the crystallinity after the printing process, which is visible on the chart (see Figure 5D). The measurements clearly indicate a visible increase in the crystalline phase of PLA for all types of composites (≈30%), but the best result is clearly observed for the PLA/5MMT sample (≈40%). In addition to the increase in crystallinity for the selected samples, it is also worth noting that the content of the crystalline phase for pure PLA is also high and reached 18%. Usually for FDM printed PLA, the crystallinity level does not exceed 10% [30]. Such a high level of crystallinity for unmodified PLA suggests that the addition of fillers is less important, and the observed increase in the content of the crystalline phase may result from a decrease in the molecular weight of PLA after the double extrusion process. Nevertheless, the results show that the behavior of the samples with the addition of 5% MMT is slightly different from the other materials. The cooling thermograms again indicate a slightly different nature of the samples with the highest addition of MMT. For most materials, a typical nucleation effect is observed, the area under the T_c_ peak is increasing and the peak maximum is shifted to higher temperatures, comparing to the reference 97 °C for pure PLA. The most intense changes are observed for PLA/20Talc and PLA/10Talc samples, where the peak intensity was the highest and the crystallization temperature was shifter over 115 °C. For BC-based samples the T_c_ peak shift was not significant; however, the area under the curve suggests improvement is possible in the crystalline phase content. An interesting phenomenon can be observed for MMT particles, where the increasing content of nanofillers indicates the reduction of the nucleation effect, as both the size of the T_c_ peak and its location for the PLA/5MMT sample are very close to the reference values. The differences observed during the cooling stage confirm the results of the previous work on the modification of PLA towards the nucleation of the crystalline phase. Usually, talc is considered to be the effective nucleating filler [40,43,59,60]. However, it is worth mentioning that the most effective PLA nucleating systems are usually materials based on an aromatic sulfonate derivative (LAK) [61,62,63]. 

The results of the second heating cycle reveal a very interesting reversal of the crystallinity change trends. As a result of the slow crystallization process carried out under controlled DSC measurement conditions, the crystallinity of all samples increased significantly. Crystallinity reached 34% even for pure PLA, while for other samples range from 58 to 67% crystallinity. The appearance of the DSC curves confirms large structural changes. As for most samples, the point of inflection of the curve around the T_g_ transition area disappears, which for polyesters is a sign of a decrease in the content of the amorphous phase. Even more pronounced signal of the material conversion to a crystalline form is the lack of the cold crystallization peak, which is direct evidence of the dominant nature of crystalline PLA in the structure. The only exception from this trend is the PLA/5MMT sample, where the increase in crystallinity was less clear and visibly lower than for the other composite samples, reaching only 49%. This behavior is a rather unexpected case where the efficiency of the filler application is higher in the case of quench cooling, rather than slow/controlled colling. This rather unusual behavior is a consequence of using a large amount of nano modifier or successful exfoliation of the filler particles. In such systems, the dispersion of the filler particles leads to a limitation of the amorphous phase mobility and the polymer chains are bounded on the filler surface. In normal conditions, an amorphous phase of this type is called rigid amorphous phase (RAF). RAF would be located between the lamellae of the crystalline phase of PLA. This phenomenon was already described for PLA by several authors [64,65,66]. Here an additional amount of polymer chains is located between the exfoliated filler particles, which increases its total content and leads to a reduction in the growth of the crystalline phase. For standard fillers, the RAF content is lower, which allows for the growth of the crystalline phase within the volume of mobile polymer chains, hence the greater ability to crystallize under favorable cooling conditions. 

## 4. Discussion and Conclusions

The analysis of the material’s properties showed different reinforcing efficiency of the used fillers. Two compositions stand out from the prepared samples—PLA with 20% of talc particles and PLA with 5% MMT. The addition of these fillers will have a positive effect not only on the stiffness and strength of the samples but also on their impact strength. In particular, the properties of MMT fillers revealed to be the most favorable since the overall filler content was relatively small. 

The addition of 10% and 20% milled biocarbon increased the stiffness of the material, but its addition had a negative influence on impact strength—thus making the composites more susceptible to brittle fracture. Taking into account the already low impact strength of pure PLA, the use of this filler is disadvantageous. The results partially confirm the previous attempts to use BC to modify PLA. Numerous studies indicate a negligible strengthening effect and the need to hybridize the structure with fibrous reinforcement [40,67,68]. 

Similar observations concern the results of thermomechanical measurements. The DMTA analysis shows differences in the stiffness of the samples in the initial measuring range, but close to the glass transition region, the modulus differences lose their importance, and the stiffness of the samples drops rapidly. Such a course of viscoelastic properties suggests that the thermal resistance of materials after the printing process will still be at a level similar to the PLA reference results. The results of DSC measurements suggest the possibility of increasing the content of the crystalline phase; however, an annealing procedure will be necessary [22,69,70,71]. For the tested materials, it would be possible already at 100 °C, but in the perspective of using this method on ready-made models with a more complex geometry than standardized ISO test samples, the softening process would certainly lead to permanent deformation of the part’s geometry. Presumably the printed parts would buckle first and then harden as a result of the increase in the crystalline phase content. As part of future research, it is planned to carry out work aimed at developing composite systems with sufficient stiffness to carry out the annealing procedure. Initial plans assume the use of a hybrid system containing nano and micrometric particles.

As confirmed by the numerous conducted analyses, composite containing 5% MMT was characterized by the best mechanical and thermomechanical properties. Therefore, future research will be focused on increasing the percentage of this filler. It will be checked whether a further increase in the content of this filler will still have a positive effect on the properties of the developed blend. The second important direction of work will be the use of polymer blend systems. In this case the main focus of the work will be the need to obtain higher impact toughness.

## Figures and Tables

**Table 1:** The sample designations and material formulations for the prepared composites.

Sample	MMT [%]	BC [%]	Talc [%]
PLA	-	-	-
PLA/1MMT	1	-	-
PLA/2MMT	2	-	-
PLA/5MMT	5	-	-
PLA/10BC	-	10	-
PLA/20BC	-	20	-
PLA/10Talc	-	-	10
PLA/20Talc	-	-	20

**Table 2:** The results of density/porosity calculations and measurements.

Sample	Density (Calculated) [g/cm^3^]	Density (of Filament) [g/cm^3^]	Density (of FDM Part) [g/cm^3^]	Porosity (of FDM Part) [%]
PLA	1.25 *	1.247 (±0.005)	1.203 (±0.034)	3.9
PLA/1MMT	1.26	1.245 (±0.015)	1.214 (±0.031)	3.6
PLA/2MMT	1.26	1.246 (±0.008)	1.218 (±0.047)	3.3
PLA/5MMT	1.28	1.269 (±0.011)	1.231 (±0.010)	3.8
PLA/10BC	1.28	1.267 (±0.014)	1.235 (±0.021)	3.5
PLA/20BC	1.31	1.297 (±0.009)	1.261 (±0.042)	3.7
PLA/10Talc	1.32	1.313 (±0.011)	1.278 (±0.038)	3.2
PLA/20Talc	1.41	1.394 (±0.007)	1.337 (±0.054)	5.2

* Pure PLA density was determined using injection molded sample made from the same resin type.

## Supplementary Materials

The following supporting information can be downloaded at: https://www.mdpi.com/article/10.3390/ma15155205/s1, Table S1: The mechanical properties of FDM printed samples; Table S2: The data obtained during the DSC measurements.
