# Supplementary material for: The Use of Nanoscale Montmorillonite (MMT) as Reinforcement for Polylactide Acid (PLA) Prepared by Fused Deposition Modeling (FDM)—Comparative Study with Biocarbon and Talc Fillers
Source: Materials (Basel). 2022 Jul 27;15(15):5205. doi: 10.3390/ma15155205 (PMC9369621; doi:10.3390/ma15155205)
Supplement: Supplementary file 1 [file materials-15-05205-s001.zip › materials-1804199-supplementary.pdf]

Supplementary information section

The Use of Nanoscale Montmorillonite (MMT) as Reinforcement for Polylactide Acid (PLA) Prepared by Fused Deposition Modeling (FDM)—Comparative Study with Biocarbon and Talc Fillers

Jacek Andrzejewski [1,2,*], Mateusz Markowski [2,3] and Mateusz Barczewski [1]

[1] Polymer Processing Division, Institute of Materials Technology, Faculty of Mechanical Engineering, Poznan University of Technology, Piotrowo 3 Str., 61-138 Poznan, Poland; mateusz.barczewski@put.poznan.pl
[2] MATRIX Students Club, Polymer Processing Division, Institute of Materials Technology, Poznan University of Technology, Piotrowo 3 Str., 61-138 Poznan, Poland; mateusz.m.markowski@student.put.poznan.pl
[3] Faculty of Materials Engineering and Technical Physics, Poznan University of Technology, Piotrowo 3 Str., 61-138 Poznan, Poland
* Correspondence: jacek.andrzejewski@put.poznan.pl; Tel.: +48-61-647-5858

Table S1. The mechanical properties of FDM printed samples.

	Tensile Test			Flexural Test		Charpy Test
	Modulus	Strength	Elongation at Break	Modulus	Strength	Impact Strength
Sample	[MPa]	[MPa]	[%]	[MPa]	[MPa]	[kJ/m²]
PLA	2780 (±36)	39.4 (±0.9)	1.9 (±0.1)	2700 (±115)	92.7 (±3.0)	2.5 (±0.2)
PLA/1MMT	2120 (±44)	29.2 (±0.2)	2.7 (±0.3)	2090 (±64)	69.5 (±3.1)	3.0 (±0.6)
PLA/2MMT	2400 (±187)	33.5 (±1.7)	2.8 (±0.1)	3040 (±119)	95.4 (±5.8)	2.4 (±0.4)
PLA/5MMT	3470 (±18)	57.2 (±1.1)	3.1 (±0.2)	3800 (±393)	129.2 (±10.5)	3.8 (±0.1)
PLA/10BC	2410 (±55)	54.6 (±1.1)	2.1 (±0.1)	3700 (±240)	150.9 (±8.8)	2.0 (±0.2)
PLA/20BC	3790 (±50)	54.7 (±0.5)	1.7 (±0.1)	4230 (±146)	134.6 (±8.7)	1.6 (±0.3)
PLA/10Talc	3659 (±95)	54.1 (±0.6)	2.9 (±0.1)	4120 (±60)	135.8 (±1.1)	1.8 (±0.2)
PLA/20Talc	4170 (±56)	57.1 (±0.3)	2.2 (±0.1)	4840 (±110)	163.4 (±1.8)	2.9 (±0.3)

Table S2. The data obtained during the DSC measurements.

	Peak position [°C]		Enthalpy [J/g]		Crystallinity (content) *	Crystallization peak
Sample	Cold cryst.	Melting	Cold cryst.	Melting	[%]	[°C]
PLA	105.1	169.1	36.9	53.9	18.2	97.0
PLA/1MMT	98.1	167.3	31.4	59.8	30.6	102.3
PLA/2MMT	98.5	167.1	31.4	57.7	28.6	104.4
PLA/5MMT	92.0	165.8	23.8	59.9	40.6	97.1
PLA/10BC	98.2	166.9	29.7	53.4	28.1	103.1
PLA/20BC	97.1	165.6	27.4	48.4	28.1	99.0
PLA/10Talc	93.2	166.6	28.6	52.4	28.2	115.4
PLA/20Talc	92.7	168.1	28.8	54.6	34.4	117.0

* crystallinity calculated from the 1st heating stage
